# Shared decision making and the practice of community translation in presenting a pre-final Afrikaans for the Western Cape Disabilities of the Arm, Shoulder and Hand (DASH) questionnaire: a proposal for improved translation and cross-cultural adaptation

**DOI:** 10.1186/s41687-019-0144-z

**Published:** 2019-08-14

**Authors:** Susan de Klerk, Christina Jerosch-Herold, Helen Buchanan, Lana van Niekerk

**Affiliations:** 1 Present Address: Division of Occupational Therapy, Department of Health and Rehabilitation Science, Faculty of Medicine and Health Science, Stellenbosch University, Cape Town, South Africa; 2 Present Address: School of Health Sciences, University of East Anglia, Norwich, UK; 3 Present Address: Division of Occupational Therapy, Department of Health and Rehabilitation Sciences, Faculty of Health Sciences, University of Cape Town, Cape Town, South Africa; 4 Present Address: Division of Occupational Therapy, Department of Health and Rehabilitation Science, Faculty of Medicine and Health Science, Stellenbosch University, Cape Town, South Africa

**Keywords:** Cross cultural translation and adaptation, Community translation, Shared decision making, Patient reported outcome measures

## Abstract

**Background:**

Translation and cross cultural adaptation of patient reported outcome measures (PROMs) involves a step referred to as harmonisation, following forward and backward translation of the measure. This article proposes the introduction of methods not previously included in the process of harmonisation. The aim of the study was to introduce shared decision making (SDM) and the practice of community translation (CT) during the harmonisation of the Afrikaans for the Western Cape version of the Disabilities of the Arm, Shoulder and Hand (DASH) questionnaire, a PROM that measures symptoms and activity and participation in persons with upper limb conditions.

**Methods:**

A broader approach to harmonisation is proposed by incorporating CT and SDM in addition to existing methods toward harmonisation. Participants (*n* = 8) involved in the harmonisation meeting included the principal investigator, a linguistic expert, occupational therapists with knowledge of the target population, context and the DASH questionnaire and members of the target population with and without upper limb conditions. A partnership was formed with the participants (a principle of SDM) and the principles of *non-parallel CT* and the *CT approach* were applied during harmonisation. Employing CT principles ensures that the norm for the translation is set by the population the translation is intended for.

**Results:**

Forward and backward translation of the DASH questionnaire presented a version of the measure in the target language for consideration during harmonisation. There were however a significant number of conceptually problematic items on the version presented at the meeting. Only seven items (7 of 30) remained unchanged.

**Conclusion:**

SDM and CT was used during the harmonisation of the Afrikaans for the Western Cape DASH questionnaire. Both these practices could have relevance in the translation and cross-cultural adaptation of PROMs where the translation is intended for persons from low socio-economic backgrounds and low levels of education.

**Electronic supplementary material:**

The online version of this article (10.1186/s41687-019-0144-z) contains supplementary material, which is available to authorized users.

## Background

Translation and cross cultural adaptation of patient reported outcome measures (PROMs) is a necessary and important step toward clinical utility in countries and contexts other than the one in which the PROM was developed. In this article the novel use of community translation (CT) together with a shared decision making (SDM) approach is proposed as a method to improve translation and cross cultural adaptation. The translation and cross cultural adaptation of the Disabilities of the Arm, Shoulder and Hand (DASH) questionnaire will be used as an example to explain the method.

Community translation is an emerging subfield within translation studies. It is defined as the translation of text towards improved communication between persons without good command of mainstream language(s) and those working in the public service [1], such as health service providers. In CT the norm for the translation is set by the population the translation is intended for. Involvement of members of the population in the translation is preferred. The *CT approach* and *non-parallel CT* are types of CT approaches [2, 3]. *Non-parallel CT* implies that the target populations of the source text and the new language version are not on parallel literacy levels [2, 3]. *Non-parallel* translation aims to simplify text and include the use of para-texts (additions to the main text in order to highlight meaning) [2]. The *CT approach* assumes that there may be similarities between the target populations of the source and the translated versions, but the intention is to have a simplified translation [2, 3]. This is done by using simple language, short sentences, avoiding passive voice sentences and addressing the reader directly [2]. As an advocate of CT approaches Lesch argues that translation is embedded within a specific context [2]. Therefore the translation has to be done in such a way that it ties in with the contextual experience of the receiver of the translation, i.e. the target population [2].

Shared decision making is understood to be a component of evidence based practice [4]. At its core SDM is about incorporating the patients’ values and preferences in decisions that affect them. Légaré and Witteman state that cultural factors and factors affecting “patient-clinician” interactions such as trust and similarities or differences in language are important factors in SDM [5]. Much has been written about the introduction and measurement thereof (SDM) within the medical fraternity [6–8]. Most interestingly, a recent systematic review explored how the development of PROMs towards evaluating the outcome of SDM in clinical practice did not routinely involve patients in the development thereof [9]. SDM is traditionally applied in the interaction between health care professionals and patients as a collaborative effort towards making decisions about their health [10]. The concept of SDM is underpinned by the understanding that the patient has the right to be informed about their options and to choose the option most important to them based on their values and preference [5]. PROMs (such as the DASH) measure the outcome of such decisions as it relates to quality of life, symptoms and/or function [11]. Even though the principles of SDM may be applied in such interactions, there is a lack of evidence of patient involvement in the development or translation and cross-cultural adaptation of measures to evaluate the outcome of the decided intervention. We hypothesise that the introduction of SDM and CT during harmonisation of the DASH could lead to improved translation and cross cultural adaptation.

Developers of PROMs provide guidelines for translation and cross cultural adaptation which usually involve an iterative process of forward and backward translation followed by harmonisation and pretesting or cognitive interviewing (CI). Societies such as the International Society for Pharmacoeconomics and Outcomes Research (ISPOR) present principles of good practice for translating and culturally adapting PROMs [12]. Researchers can also consider definitions and items from resources such as the COnsensus-based Standards for the selection of health Measurement INstruments (COSMIN) checklist when designing and conducting translation and cross cultural adaptation studies [13]. Similarly, the Patient-Reported Outcome Measurement Information System (PROMIS) offers standards for language translation and cultural adaptation based on the Functional Assessment of Chronic Illness Therapy (FACIT) translation methodology chart [14]. The present study concerns the translation and cross cultural adaptation of the DASH questionnaire into Afrikaans for the Western Cape (South Africa). The DASH questionnaire is a 30 item PROM, developed in 1996 by the Institute for Work and Health (IWH) (in Canada) as a measure of activity and participation, symptoms and disability in persons with upper limb conditions (in accordance with the International Classification of Functioning, Disability and Health) [15]. The DASH is used extensively in research and clinical practice by occupational therapists, physiotherapists and surgeons treating persons with upper limb conditions and has been translated into more than 50 languages across the world. This article reports on a component of a broader study that aims to translate and cross culturally adapt the DASH into Afrikaans for the Western Cape and evaluate the psychometric properties including content and structural validity, and clinical utility.

**Fig. 1:**
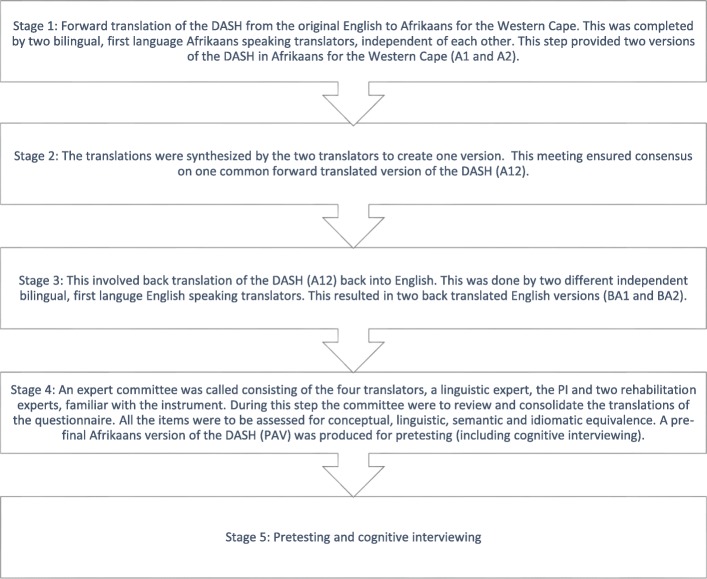
Stages of translation and cross-cultural adaptation recommended for approval of a translated version of the DASH by the IWH

Clear guidelines are provided for the translation and cross-cultural adaptation of the DASH into a new language version [16]. The guidelines by Beaton, Bombardier, Guillemin and Ferraz, freely available from the DASH website, outline the five stage process for the translation and cross cultural adaptation of the DASH (Fig. 1). The principal investigator (PI) (and 1st author) communicated the intent to translate and cross culturally adapt the original English source version of the DASH to Afrikaans for the Western Cape. The stages of translation and cross-cultural adaptation recommended for approval of a translated version of the DASH by the IWH were carefully followed. Stage 1 to 3 were completed as per the recommendations. Stage 4 entails an expert review committee [16]. Beaton et al. recommend a panel consisting of the following individuals: the PI, the four translators (involved in the forward and back translation of the instrument), a linguistic expert, and two rehabilitation experts familiar with the instrument [16]. During this stage the committee is to review and consolidate the translations of the questionnaire. All the items must be assessed for conceptual, linguistic, semantic and idiomatic equivalence [16]. A pre-final version of the DASH is then produced for pretesting (including Cognitive Interviewing) in stage 5. It has however been reported that these expert committee reviews (stage 4) may generate a sample unrepresentative of the target population [9, 17]. In addition, authors have highlighted that the physical setting in which these take place is often a different environment from the setting in which the instrument will be administered [9, 18]. The target population in the present study consists of Afrikaans speaking individuals from low socio-economic backgrounds within the Western Cape of South Africa. Afrikaans is spoken by 13.5% of the population of South Africa and most widely used in the Western and Northern Cape of South Africa [19]. Blignaut highlights that there is great variation in the Afrikaans language that is “*fed by differing social, cultural, geographical, situational and psychological contexts*” [20], p 20. Lesch, in offering a CT approach, does not define the group (the population for whom the translated DASH is intended) as a political entity, linked to colour or race, but as a grouping of persons from a specific socio-economic background, juxtaposed with middle to high income groups [2]. This group can be further delineated by the fact that the majority are public health service users with low levels of education and little to no post school qualification. Many are not medically insured, generally unemployed and on low paying contract positions. A dialectic Afrikaans is spoken, different to standard Afrikaans, where little attention is paid to language and the use of simple language is the norm. Persons are described to be language impoverished [2, 3]. Characteristics of the language are dialectical use of language and code switching between non-standard Afrikaans and English [2, 20, 21]. In addition, written communications may be avoided through fear of not being able to communicate effectively [2].

The objective of this article is to propose the introduction of CT (specifically *non-parallel CT* and the *CT approach*) and SDM during Stage 4 (harmonisation) of the translation and cross cultural adaptation of the DASH. Reporting on the harmonisation of the Afrikaans for the Western Cape DASH will be used as an example to illustrate the approach.

## Methods

Wild et al. suggests two possible ways of achieving harmonisation following back translation of items [12]. The first involves the provision of verbal back translation of each PROM item, which is then carefully considered against the source version. In the second, which was the favoured approach in the current research, the PI (1st author) identifies items that could be conceptually problematic and shares translation solutions with the committee. As a result of the introduction of a SDM approach during harmonisation patients and healthy participants from the target population were included, within the target setting in addition to the individuals suggested by Beaton et al. [16] and Wild et al. [12]. In CT the receiver (the population for whom the translation is intended) sets the norm for the translation [2]. The PI therefore encouraged translation solutions to be offered by the committee members. Committee members were encouraged to review instructions, items and response options in order to ensure conceptual equivalence. Any occurring construct mismatch was corrected and preferences of persons from the target population were used for item wording.

### Research context – population and setting

The research took place in the context of Bishop Lavis on the Cape Flats, more specifically the Bishop Lavis Rehabilitation Centre. Bishop Lavis is a township in the Northern Suburbs of Cape Town that spans an area of 2.58 km^2^ [22]. It was established under apartheid and has a population of 26,482 (10,247.48 per km^2^) people across 5788 (2239.73 per km^2^) households, in both formal and informal housing structures [22, 23]. Poverty and low levels of education and employment prevail in Bishop Lavis*.* The majority of people are Afrikaans speaking (86.45%) [22]. The Bishop Lavis Rehabilitation Centre serves the community as a public primary level health care institution. Rehabilitation is offered to community members with a range of conditions including but not limited to upper limb (UL) conditions.

### Harmonisation meeting

The meeting took place in a therapy room within the Bishop Lavis Rehabilitation Centre on Friday 3 August 2018 from 9:00 until 12:00. Participants (Table 1) were purposively sampled based on recommendations by Beaton et al., i.e. previous involvement in the translation process (forward and back translators); linguistic expertise; being an occupational therapist with knowledge of the target population, context and the DASH questionnaire [16]. In addition, due to the introduction of CT and SDM approaches members of the target population with and/or without upper limb conditions were also recruited to participate. The translators involved in the forward and backward translation of the DASH were however unable to attend. Extensive notes made by the translators, were made available following their synthesis after the back translation was completed. These notes were found sufficient in the discussion during the harmonisation meeting.

**Table 1:** Participants in harmonisation meeting

Number	Participant	Description
1	Principal investigator	Key in-country person [1]
2	Linguistic expert	Academic who studies language
3 and 4	Occupational therapist	Rehabilitation expert
5 and 6	Patient with upper limb condition	Member of target population
7 and 8	Volunteer at Bishop Lavis Rehabilitation Centre	Member of target population

### Data collection and instrumentation

Participants received copies of the source version of the DASH, the forward translated and the back translated versions of the Afrikaans for the Western Cape DASH and a table outlining some pre-identified items requiring adjustment. During the harmonisation meeting the PI made extensive notes as the meeting progressed. Participants were also encouraged to make notes as the meeting progressed, which were collated within the analysis. The meeting was audio recorded to refer back to in case any information was missed during note keeping. Equal participation was encouraged and facilitated by the PI. Participants were asked to comment on the instructions, test items and layout of the pre-final Afrikaans for the Western Cape DASH (the DASH-PAV). Principles of SDM were applied during the harmonisation meeting. These included developing a partnership; establishing or reviewing participants’ role in decision making; ascertaining and responding to ideas, concerns and expectations; identifying choices and evaluating those against the source and translated versions of the DASH; reflecting upon and assessing the impact of alternative decisions; and, making a decision in partnership [4]. In addition this allowed for the consideration of the principles of CT in the collation of the DASH-PAV.

## Results

The results of the harmonisation meeting are presented in terms of the DASH-PAV items requiring adjustment, solutions, the rationale and principles of CT (both *non-parallel CT* and the *CT approach*) as they were considered during the meeting. Even though the forward and back translation presented a version of the questionnaire in the target language, there was a significant amount of conceptually problematic items on the version presented at the harmonisation meeting. Only seven items (of 30) remained unchanged. Additional file 1 details the conceptually problematic translations and the solutions that were either presented by the PI or derived through the conversation and interaction with the participants of the meeting. The rationale for each solution is also provided in Additional file 1. Several principles underpin each of the decisions regarding the adaptation of the item or instruction wording; these are summarised in Table 2.

**Table 2:** Principles of community translations applied to test items

Item	Principles							
	Code switching [2, 20]	Non-parallel community translation: para text [2]	Non-parallel community translation: simplify text (colloquial or dialectic use of language) [2, 3]	Community translation approach: using simple language [2, 3]	Community translation approach: short sentences [2]	Community translation approach: don’t use cumbersome concepts [2]	Community translation approach: Avoid use of passive voice [2]	Community translation approach: address the reader directly [2, 3]
Title of the questionnaire: Disabilities of the arm shoulder and hand.	✓		✓					
Instruction on first page				✓	✓	✓		✓
Instruction page for first 21 test items:			✓			✓		✓
Wording of five point Likert scale for items 1 to 21			✓					
Item 1: Opening a tight or new jar.			✓			✓		
Item 3: Turning a key				✓				
Item 6: Place an object on a shelf above you head.			✓					
Item 7: Source version: Do heavy household chores (e.g., wash walls, wash floors).				✓				
Item 8: Garden or do yard work	✓		✓					
Item 9: Make a bed		✓	✓					
Item 10: Carry a shopping bag or briefcase	✓		✓	✓				
Item 11: Carry a heavy object (over 10 lbs)				✓		✓		
Item 12: Change a lightbulb overhead	✓	✓						
Item 13: Wash and blow dry your hair						✓		
Item 14: Wash your back						✓		
Item 15: Put on a pullover sweater.	✓							✓
Item 17, 18 and 19: Relating to recreational activities	✓					✓	✓	
Item 20: Manage transportation needs (getting from one place to another).	✓			✓		✓		
Item 21: Sexual activities			✓					
Wording of five point Likert scale for items 22 to 29			✓					
Item 22: During the past week, to what extent has your arm, shoulder or hand problem interfered with your normal social activities with family, friends, neighbours or groups?	✓			✓	✓			✓
Item 23: During the past week were you limited in your work or other regular daily activities as a result of your arm, shoulder or hand problem?				✓	✓		✓	✓
Item 26: Tingling (pins and needles) in your arm, shoulder or hand.	✓	✓						
Item 28: Stiffness in your arm, shoulder or hand.	✓	✓						
Item 29: During the past week, how much difficulty have you had sleeping because of the pain in your arm, shoulder or hand?							✓	✓
Item 30: I feel less capable, less confident or less useful because of my arm, shoulder or hand problem.	✓		✓					
Work module and Sport and Performing arts Module						✓		

## Discussion

This article proposes the introduction of CT (specifically *non-parallel CT* and the *CT approach*) and SDM during Stage 4 (harmonisation) of the translation and cross cultural adaptation of the DASH through reporting on the harmonisation of the Afrikaans for the Western Cape DASH. An important underpinning of both CT and SDM is the incorporation of patient opinion. Barr and Elwyn advocate the use of cognitive interviewing (CI) as a means to incorporating patient opinion [9]. Willis presents a ten step sequence of the questionnaire development process in which the evaluation of the questions through CI is preceded by an “*expert appraisal of questions for common pitfalls*” [24], p.137. The report of the ISPOR Task Force for Translation and Cultural adaptation outlines principles of good practice for the translation and cultural adaptation process for PROMs [12]. In this publication the process referred to as *harmonization* is introduced prior to CI. The authors state that harmonisation is aimed towards detecting and dealing with translation discrepancies between different language versions and involves researchers and language experts [12]. It is suggested that not doing this step could lead to translations that are different between language versions [12]. The authors acknowledge that this step requires further investigation and is often omitted from translation and cross cultural adaptation guidelines, as is the case with the IWH guidelines for the DASH. The DASH was developed as a self-report questionnaire towards measuring physical function and symptoms in individuals with upper limb conditions. If the cross cultural adaptation of the measure is to be embraced, the argument is made that the patient is as much an expert as the translators, linguistic or rehabilitation experts, able to appraise the test items and consider their preferences and values against their social and cultural background. In considering cross-cultural validity of PROMs, the COSMIN checklist enquires about the expertise of the persons involved in the translation, for example, their expertise regarding the specific condition and/or disease and their expertise in the construct purported to be measured by the PROM [13]. We argue that patients and/or healthy individuals from the target population can be considered experts on the condition or disease as well as the construct to be measured. Introduction of the practice of CT and principles of SDM during harmonisation in the cross cultural adaptation of the Afrikaans for the Western Cape DASH questionnaire allowed for inclusion of persons from the target population.

*Non-parallel CT* implies that the target populations of the source text (the original DASH developed in English in the developing context of Canada) and the DASH for the Western Cape are not on parallel literacy levels [2, 3]. *Non-parallel CT* therefore aimed to simplify text and included the use of para-texts (additions to the main text in order to highlight meaning) [2] (Additional file 1). In addition the *CT approach* was also considered during the harmonisation of the DASH-PAV [2, 3]. The *CT approach* assumed that there may be similarities between the target populations of the source and the translated versions, but the intention was to have a simplified translation [2, 3]. This was done by using simple language, using short sentences, avoiding passive voice sentences and addressing the reader directly [2] (Additional file 1).

In the present study, the harmonisation took place within the context of Bishop Lavis and a partnership was developed (a principle of SDM) by communicating the intent to have involvement from persons representative of the target population [4]. The DASH items include measuring a range of activities and participation in life situations. The activities and participation in life situations relates to the specific context and culture, an understanding of which cannot be derived by professional translators, linguistic or rehabilitation experts, who do not form part of the context or culture. In addition to the differences in context and culture between the suggested panel members for the harmonisation of the DASH -PAV [16] and the target population, there is the issue of a translation that could be inaccessible as a result of the target populations’ language impoverished status as outlined above, now confronted with a translation by persons with good command of the language. The absence of the forward and backward translators from the harmonisation meeting could be considered a limitation in translation and cross cultural adaptation studies. However, we argue that the limitation relates to the fact that the forward and backward translators did not take a CT approach during the translation of the DASH into Afrikaans for the Western Cape. Care was therefore taken to identify choices (a principle of SDM) and evaluate these against the source and translated versions of the DASH-PAV. It is evident from the suggested changes captured in Additional file 1 and the application of principles of CT across test items (Table 2), that context and culture were considered in the translation. In addition the role of all participants in the harmonisation meeting, in the decision making [4] with regards to the translation of test items were made explicit, which led to an appropriate translation, fit for the intended population. As a continuation of the broader study, the pre-final version of the Afrikaans for the Western-Cape DASH will be further evaluated through pre-testing and cognitive interviewing, outlined in stage 5 in the process of translation and cross cultural adaption of the DASH [16]. A recommendation for future studies is the comparison of a PROM, translated into one target language independently by two teams, with one using SDM and CT during harmonisation and the other not. The resulted translations could be reviewed and compared by a sample from the target population on aspects of conceptual, linguistic, semantic and idiomatic equivalence to assess which method results in a better translation and cross-cultural adaptation.

## Conclusion

SDM and CT were introduced during the harmonisation of the Afrikaans for the Western Cape DASH questionnaire. We propose that both these practices could have relevance in the translation and cross-cultural adaptation of PROMs where the translation is intended for persons from low socio-economic backgrounds and low levels of education; and where variation exists between the translators and the population for whom the translation is intended.

## Additional file

Additional file 1: Items requiring adjustment and rationale and solutions for adjustment. (DOCX 25 kb)

## Data Availability

Data analysed during this study are included in this published article [and its additional information file]. Full data sets are available from the main author upon reasonable request.

## Abbreviations

CI: Cognitive interviewing
COSMIN: The consensus-based standards for the selection of health status measurement instruments
CT: Community translation
DASH: Disabilities of the Arm, Shoulder and Hand Questionnaire
DASH-PAV: Pre-final Afrikaans for the Western Cape Disabilities of the Arm, Shoulder and Hand Questionnaire
EBP: Evidence based practice
FACIT: Functional Assessment of Chronic Illness Therapy
ICF: International Classification of Functioning, Disability and Health
ISPOR: International Society for Pharmacoeconomics and Outcomes Research
IWH: Institute for Work and Health
PI: Principal Investigator
PROMIS: Patient-Reported Outcome Measurement Information System
PROMs: Patient reported outcome measures
SDM: Shared decision making
UL: Upper limb
