# Supplementary material for: Shared decision making and the practice of community translation in presenting a pre-final Afrikaans for the Western Cape Disabilities of the Arm, Shoulder and Hand (DASH) questionnaire: a proposal for improved translation and cross-cultural adaptation
Source: J Patient Rep Outcomes. 2019 Aug 14;3:52. doi: 10.1186/s41687-019-0144-z (PMC6692802; doi:10.1186/s41687-019-0144-z)
Supplement: Supplementary file 1 — Items requiring adjustment and rationale and solutions for adjustment. (DOCX 25 kb) [file 41687_2019_144_MOESM1_ESM.docx]

Additional file 1: Items requiring adjustment and rationale and solutions for adjustment

| **Item requiring adjustment** | **Rationale / Solution** |
| --- | --- |
| Title of the questionnaire: Disabilities of the arm shoulder and hand. | The DASH intends to measure symptoms and the impact of the arm or hand condition on functioning. The version presented at the harmonisation meeting had the word *besering* [injury] in the title as a translation of *Disabilities.*  *Disabilities* does not translate to *beserings* [injury], which does not encompass all the conditions that could be present in the upper limb, over and above injury. The accepted translation of disability, *gestermdheid* is problematic as it is not a word that is considered politically correct in the health sciences. The suggestion from the group was to keep the English word *Disabilities* in the title of the questionnaire. Code-switching is offered as a rationale but more so the fact that the members of the target population, represented at the meeting, felt that this is a word people will understand well, over and above any Afrikaans translation of the word *disability.* The title of the questionnaire was therefore changed to: *Disabilities van die arm, skouer en hand.* [Disabilities of the arm, shoulder and hand] |
| Instruction on first page | To avoid passive voice as per the community translation approach the instruction: *Beantwoord asseblief elke vraag deur ‘n sirkel om die nommer te trek wat jy kies.* [Answer every question by circling the number you choose] was changed to: *Beantwoord asseblief elke vraag. Trek ‘n sirkel om die nommer wat jy kies.* [Answer every question. Circle the number you choose]. Similarly, the instruction: *Gee ‘n skatting van hoe maklik of hoe moeilik jy ‘n aktiwiteit sal kan uitvoer* [Give and estimation of how easy or difficult you would find doing an activity] was changed to: *Skat hoe maklik of hoe moeilik jy ‘n aktiwiteit sal kan uitvoer.* [Estimate how easy or difficult you would find doing an activity]  In order to simplify the text and avoid misinterpretation, the last part of the instruction namely: *Dit maak nie saak watter arm of hand jy gebruik om die aktiwiteit te doen nie, baseer jou antwoord op jou vermoë om die aktiwiteit te doen, ongeag hoe jy dit doen.* [It doesn’t matter which arm or hand you use to do the activity, base your answer on your ability to do the activity, irrespective of how you do it.] was changed to: *Dit maak nie saak watter arm of hand jy gebruik om die aktiwiteit te doen nie; jou antwoord moet gaan oor jou vermoë om die aktiwiteit te doen. Dit maak ook nie saak hoe jy dit doen nie.* [It does not matter which arm or hand you use to do the activity, your answer should be about your ability to do the activity. It also does not matter how you do it.] This change reflects simple use of language. |
| Instruction page for first 21 test items: | The following instruction was offered by the PI at the harmonisation meeting and accepted by the group. *Trek asseblief 'n sirkel om die antwoord wat die beste by jou pas. Dink asseblief terug oor hoe dit vir jou die laaste week was wanneer jy antwoord. Hoe maklik of hoe moeilik was die aktiwiteite vir jou?* [Draw a circle around the answer that best fits with you. Please think back about how it was for you during the last week when you answer. How easy or how difficult was the activity for you? ] In this suggestion, the use of language is simplified, the reader is spoken to directly with the use of *jou* [you] and difficult terms such as *omkring* [circling] is avoided. |
| Wording of five point Likert scale for items 1 to 21 | The source version of the DASH included the following words for this section: No Difficulty, Mild difficulty, Moderate difficulty, Severe difficulty, Unable. The version presented at the harmonisation meeting included the following: *Glad nie moeilik nie, Effens moeilik, Moelik, Baie moeilik, Onmoontlik.* [ Not difficult at all, slightly difficult, difficult, very difficult, impossible] Members of the group suggested that the word *effens* [slightly] be replaced with *bietjie* [a little] as a word that would be better understood by the target population and routinely used in everyday conversation. |
| Item 1: Opening a tight or new jar. | In the version presented at the meeting ‘jar’ was translated to *fles.* The group however felt that this is not a good translation and suggested that the language be simplified by using *potjie*. The items therefore reads: ­ ‘*n Nuwe potjie of ­ potjie wat styf toegedraai is oop te maak*. |
| Item 3: Turning a key | Participants felt that translation of turning a key into Afrikaans *‘n Sleutel te draai* could be interpreted as swinging a key. This item was therefore changed to *‘n Deur oop of toe te sluit met ‘n sleutel* [To lock or unlock a door with a key] in order to make it more accessible and understandable for the reader. |
| Item 6: Place an object on a shelf above you head. | The translation offered through the process of forward and backword translation was *Iets op ‘n rak sit wat bokant jou kop is,* a direct translation of source version. The language was however simplified by changing the item to: *Iets op ‘n rak sit wat hoër as jou kop is.* [To put something on a shelf that is higher than you head ] |
| Item 7: Source version: Do heavy household chores (e.g., wash walls, wash floors). | The translation presented at the meeting did not include the translation of doing heavy household chores. The PI therefore suggested this be added (*Om baie harde huiswerk te doen*). A further consideration was whether washing walls and floors are indeed considered heavy household chores in this context, as seen in the example of the Yoruba DASH.^19^ Participants added the household task of moving furniture in addition to washing walls and floors. |
| Item 8: Garden or do yard work | The translation *Tuin skoonmaak of buite werk* [Cleaning garden or working outside] was not thought to suffice. As many do not have a garden it was thought to lead this question with cleaning the yard, as people are more likely to have a small yard around the house than a garden. Furthermore, the word *yard* was preferred above the Afrikaans translation *agterplaas,* introducing code switching and simplifying the language. The item therefore reads: *Yard skoonmaak of in die tuin werk.* |
| Item 9: Make a bed | The word *kooi* a colloquial reference to a bed replaced bed in the item and *bed* was added to the translation in brackets as per the principles of non-parallel community translation (adding a para-text). |
| Item 10: Carry a shopping bag or briefcase | The translation of briefcase was not added to the Afrikaans version of this item, as persons are unlikely to carry briefcases in this context. Participants commented on how unsafe it would be in the context of Bishop Lavis (or other areas on the Cape Flats) to carry briefcases or suitcases as they could easily get robbed. Even shopping done in small quantities in order to safely travel on public transport and reduce the possibility of being robbed. The translation offered namely: *‘n Sak met inkopies dra* [Carry ‘n shopping bag] was considered formal use of language. Participants suggested the use of the diminutive form of *sak* [bag] namely: *sakkie* and the use of the word *groceries* by motivating this translation reflects the everyday language of the target population. |
| Item 11: Carry a heavy object (over 10 lbs) | In the Afrikaans translation the measure was changed to metric and reflected 5 kg’s. Participant however reported it would be more useful to add a specific reference of a familiar object, people are likely to pick up and carry, that weighs about 5kg’s. The group concurred that two packs of flour would equate this and is a likely item to be picked up to carry. The item was therefore changed to: *Iets dra wat 5 kg swaar is (soos 2 groot pakke meel).* [Carry something that is 5kg’s heavy (like two packs of flour)] |
| Item 12: Change a lightbulb overhead | The translation offered (*Lig bokant jou kop omruil)* was thought not to be simple enough. Participant also commented that regular reference is made to a *globe* rather than a *lig. Globe* and *lig* are both understood to refer to a lightbulb. Para-text was therefore added: *Om ­ globe (lig) bokant jou kop om te ruil.* |
| Item 13: Wash and blow dry your hair | Participants commented on the fact that washing and blow drying hair in this context is often done by others. In order to maintain the construct of this item the suggestion was to add the word *self* [yourself] to the translation. The item therefore reads: *Self jou hare te was en droogblaas* |
| Item 14: Wash your back | The same consideration was awarded this item per item 13 and the word *self* [yourself] was added here as well: *Self jou rug te was.* |
| Item 15: Put on a pullover sweater. | The Afrikaans translation offered the word *trui* [sweater or jersey]. Participants however felt that this would not be well understood and suggested the use of *jersey* and to add the reference to pulling it over your head. ­ ‘n *Jersey oor jou kop aan te trek.* |
| Item 17, 18 and 19: Relating to recreational activities | The group consider a new word for *ontspannigsaktiwiteite* [recreational activities] as well as the suggested activities. *Ontspanningsaktiwiteite* was thought to be too formal and the suggestion activities (through the forward and back translation process) were deemed not appropriate for the context. *Ontspanningsaktiwiteite* was changed to *Aktiwiteite vir ontspanning* [Activities to relax] towards a simpler use of language and the activities were changed to reflect more contextually relevant and appropriate activities that both men and woman may participate in.  Item 17: *Aktiwiteite vir ontspanning wat nie baie moeite is nie (soos dominoes, kaarte speel of brei).* [Activities to relax that is not a lot of effort (such as dominoes, playing cards or knitting)]  Item 18: *Aktiwiteite vir ontspanning wat ­ bietjie meer krag van die arm, skouer of hand nodig het (soos darts of pool).* [Activities to relax that requires a bit more power from the arm, shoulder or hand (such as darts or pool)]  Item 19: *Aktiwiteite vir ontspanning waar jy jou arm vrylik*  *beweeg (soos tempin bowling of foosball).* [Activities to relax that where you can move your arm freely (such as tempin bowling or foosball)]  Code switching were employed, as participants felt this would be better understood. |
| Item 20: Manage transportation needs (getting from one place to another). | As per the translation offered through the forward and backward translation process, just moving from one place to another does not require much upper limb (UL) function. Managing transport in this context does however require UL function, e.g. getting in and out of a taxi or passing money from the back to the *gaurdjie*^[[1]](#footnote-1)^ in the front. It was also felt that the translation should explore both public and private transport options. The item was therefore changed to: *Om jou eie motor of public transport te gebruik* [To use your own car or public transport]. The words *public transport* was maintained as the Afrikaans translation: *publieke vervoer* was thought to be too formal. |
| Item 21: Sexual activities | This item was discussed at great length. Participants were convinced that it had to be included in the questionnaire and thought is an important item to consider. However, the group felt that the direct translation of sexual activities reflects formal use of language and that it does not capture additional aspects of intimacy that could be affected by an UL condition. The translation offered was therefore: *Om intiem te wees.* [To be intimate] |
| Wording of five point Likert scale for items 22 to 29 | As per the wording of five point Likert scale for items 1 to 21. *Effens* [slightly] was replaced with *bietjie* [a little]. |
| Item 22: During the past week, to what extent has your arm, shoulder or hand problem interfered with your normal social activities with family, friends, neighbours or groups? | The text was simplified, sentence kept short and the reader addressed directly. The suggested translation also avoided the use of passive voice. Participant also suggested the retention of the word *neighbours* and *social life*. The items reads: *In die laaste week hoe het jou probleem met jou arm, skouer of hand ­ impak op jou social life met jou familie, neighbours of vriende gehad.* [In the past week how did your problem with your arm, shoulder of hand impact your social life with your family, neighbours or friends]. |
| Item 23: During the past week were you limited in your work or other regular daily activities as a result of your arm, shoulder or hand problem? | As per item 22 (simplified text, with short sentence, addressing the reader directly and avoiding passive voice):  *Hoe het die probleem met jou arm, skouer of hand,*  *jou op enige manier gekeer die laaste week om jou*  *daaglikse aktiwiteite of werk te doen.* |
| Item 26: Tingling (pins and needles) in your arm, shoulder or hand. | The Afrikaans translation of tingling namely *prikkelgevoel* was thought to be too formal use of language. Participants suggested the retention of *pins and needles* (code switching). Item 26 therefore reads: *Pins en needles in jou arm, skouer of hand.* |
| Item 28: Stiffness in your arm, shoulder or hand. | Stiffness was translated to *styfheid.* Participants however felt that the word *tightness* is routinely used to describe stiffness. The two participants with UL conditions reflected on they would say that a joint or an arm feels tight (meaning stiffness). Stiffness was therefore translated to *tightness,* with the inclusion of the para-text *styfheid.* Item 28: *Tightness (styfheid) in jou arm, skouer of hand.* |
| Item 29: During the past week, how much difficulty have you had sleeping because of the pain in your arm, shoulder or hand? | The translation offered through the back and forward translation was in the passive voice and therefore it was changed to: *Hoe moeilik was dit vir jou om die afgelope week te slaap met die pyn in jou arm, skouer of hand.* |
| Item 30: I feel less capable, less confident or less useful because of my arm, shoulder or hand problem. | Participants offered the continued use of the word confident in this item, as the Afrikaans translation was felt to be too formal (*selfvertroue).* Item 30: *Ek voel minder werd, minder bekwaam en minder confident as gevolg van my arm, skouer of hand probleem.* |
| Work module and Sport and Performing arts Module | The most important consideration in this section, was the layout of both modules. In the source version the blank section that prompts the reader to indicate what their job is, precedes the prompt to tick the box that reads: *I do not work (you may skip this section)*. The same applies to the sport and performing arts module. Participant thought this to be confusing and to misguide the reader. The suggestion was therefore to simplify by requiring the reader to first indicate that they do not work (and may skip this section) prior to having to indicated what job they do (in the case of them begin employed). The translation offered by the forward and backward translation process was deemed appropriate. |

1. A gaurdjie (gah-chee) is the person who calls for passengers and takes in the money on a minibus taxi. [↑](#footnote-ref-1)
